# Be Well™ Acres Homes: a community-driven, evidence-based approach to reduce health inequities through sustained cross-sector partnership

**DOI:** 10.1007/s10552-023-01818-4

**Published:** 2023-11-18

**Authors:** Ruth Rechis, Katherine B. Oestman, Michael T. Walsh, Brad Love, Ernest Hawk

**Affiliations:** 1 Cancer Prevention & Control Platform, The University of Texas MD Anderson Cancer Center, 7007 Bertner Ave. Unit 1628, 1MC13.2339, Houston, TX 77030 USA; 2 The University of Texas at Austin Center for Health Communication, Austin, TX USA; 3 Division of Cancer Prevention & Population Sciences, Office of the Vice President, The University of Texas MD Anderson Cancer Center, Houston, TX USA

**Keywords:** Population health, Risk reduction behavior, Cancer prevention, Implementation science, Cross-sector partnerships, Systems strengthening, Community outreach and engagement

## Abstract

**Purpose:**

Be Well Communities™ is MD Anderson’s signature place-based approach for cancer prevention and control, working with communities to promote wellness and address modifiable risk factors for cancer. The purpose of this paper is to describe implementation of the planning phase of the Be Well Communities model in Acres Homes which began in 2019.

**Methods:**

A community advisory group (Steering Committee) including residents, non-profit organizations, health care partners, city and county agencies, plus other stakeholders, was convened and aligned through a structured process to develop shared goals, foster multisector collaboration, as measured by a stakeholder survey administered twice, and enhance community capacity to improve health outcomes through development of a Community Action Plan.

**Results:**

Clear, achievable goals were developed, multisector collaboration was enhanced, and more than 400 h of capacity building support led to a Community Action Plan initially focused on healthy eating and active living, including 15 evidence-based interventions led by 18 organizations. The majority (93%) of the Steering Committee reports that this plan reflects community priorities and will reach the residents most in need.

**Conclusion:**

By listening and developing trust, the Be Well Communities team successfully worked with Acres Homes residents and organizations to enhance community capacity to address health inequities in one of Houston’s most diverse and historic communities.

## Introduction

Recent research estimates that as many as 50% of cancer cases could be prevented by more consistently applying current knowledge to the population [1]. Experts have routinely provided cancer prevention recommendations [2] and defined evidence-based interventions for effective cancer prevention at the community level [3, 4]. Yet, a tremendous gap continues to exist between this knowledge and the implementation of effective prevention strategies. A critical step in reducing cancer incidence is to effectively deploy evidence-based interventions for primary prevention at the population level, with a focus on communities experiencing health inequities. NCI-designated cancer centers are uniquely positioned to engage the communities they both serve and seek to serve through population health initiatives that can reduce the cancer burden and increase equitable access to the highest quality cancer prevention and control programs.

For more than 40 years, cancer prevention has been a cornerstone of The University of Texas MD Anderson Cancer Center’s mission to eliminate cancer. Established in 2016, Be Well Communities™ is MD Anderson’s place-based approach for comprehensive cancer prevention and control, by working with communities to promote wellness and address modifiable risk factors for cancer. The approach has been implemented in three Texas communities—Baytown, Pasadena, and Acres Homes. Built on nearly 100 years of healthy community initiative best practices, literature, and experience, Be Well Communities is a signature program of MD Anderson’s Cancer Prevention & Control Platform, a community impact accelerator which implements evidence-based interventions (EBIs) involving community services, public education, and policy interventions, targeting measurable reductions in cancer incidence and mortality. Be Well Communities unites individuals, schools, workplaces, government agencies, health care providers, and policymakers to plan and carry out sustainable, community-led solutions to make positive, long-lasting changes in people’s lives. Specifically working in those areas that can have a direct impact on cancer risk reduction: healthy eating, active living, sun safety, tobacco-free living, and preventive care (i.e., cancer screening and vaccines). Central to this place-based approach is creating systemic change by serving as a catalyst coordinating and supporting the implementation of EBIs through community-based organizations who will sustain the work long into the future, all with a specific focus on reducing health inequities and access issues.

### Be Well Communities Model

Be Well Communities was founded on effective strategies for community-based, cross-sector, multi-component approaches to promote health equity. Multi-component approaches can reduce risk factors such as obesity and excessive sun exposure across the lifespan [4, 5]. Successful examples of these types of approaches, such as Shape Up Somerville [6], include implementation of EBIs coupled with a community-driven coalition. Community-driven coalitions help to ensure that interventions are relevant to and executed in alignment with the needs of the community. Key components of effective community-based approaches include at a minimum three key elements: (1) making health equity a shared vision and value, (2) fostering multi-sector collaboration; and (3) increasing community capacity to shape health outcomes [7]. Through delivery of EBIs placed effectively in the context of individuals and families, community-driven coalitions can reduce disparities and improve health.

**Fig. 1:**
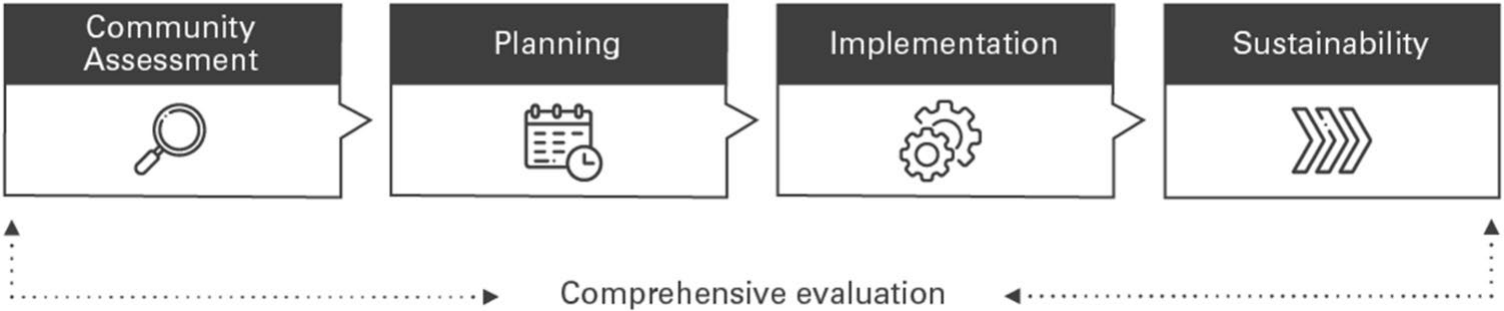
Be Well Communities Model

Leveraging these lessons, the Be Well Communities model (Fig. 1) focuses on the specific needs and context of the community through phases of community assessment, planning, and implementation, supported by evaluation and sustainability planning. An evaluation plan, developed in partnership with RTI International, guides this work to ensure adequate data collection, align organizational objectives with outcomes, and assess progress and impact.

Following the principles outlined in the Centers for Disease Control and Prevention’s (CDC) Sustainability Planning Guide for Healthy Communities (2012), sustainability is foundational and integrated into practice by creating, executing, and evaluating a Community Action Plan [8]. The model relies on three groups working collaboratively: the backbone team, a steering committee, and collaborating organizations [9]. The backbone team is responsible for overall coordination, convening, fund stewardship, management, and evaluation of the initiative. The Steering Committee, which includes community residents, is the active community advisory board or health coalition that establishes shared goals, reviews available interventions, develops the Community Action Plan, and guides and champions the initiative. Collaborating organizations are funded Steering Committee organizations carrying out EBIs tailored to the community’s culture that make up the Community Action Plan.

Additionally, since many cancer risks (e.g., tobacco exposure, obesity, sun/UVR exposure) appear to accumulate across the lifespan, it is increasingly clear that cancer prevention needs to begin in childhood [10]. As such, youth-serving institutions (e.g., elementary schools, daycares, sports programs) are critical collaborators across Be Well Communities initiatives. This includes interventions focused on vaping prevention and HPV vaccination as well as healthy eating and physical activity programming. Addressing risk factors from early childhood through adolescence, can increase healthy behaviors and reduce unhealthy behaviors, ultimately reducing the risk for cancer later in life.

Together with strong, cross-sector community partners, Be Well Communities made a tangible difference in its inaugural community, Be Well™ Baytown [11] and has since expanded that initiative and expanded work in additional communities across the Greater Houston area. More than 130,000 residents have received direct support through Be Well Communities initiatives to date. See Table 1 for additional information about Be Well Communities key indicators of short- and medium-term impact related to engagement with stakeholders across the community, investments in the built environment and integrated active living, and prioritized actions to prevent cancer and other chronic diseases. Longer-term metrics will evaluate changes in cancer-associated behaviors (e.g., HPV vaccination, sedentary behavior, tobacco use) and cancer incidence.

**Table 1:** Be Well Communities key milestones across two communities in the greater Houston area (2017–2022)

Community Capacity Building	Engagement with stakeholders across the community o 30 local, regional and state organizations are actively engaged on Be Well Communities Steering Committees, guiding the implementation of an action plan in partnership with residents in each community o 4000 + hours of capacity building, technical assistance, project management and program evaluation provided by the Be Well Communities team
Infrastructure Investments	Investments in the built environment and integrated active living o 29 sunshades have been installed at city parks, schools and college campuses o 6 walking trails built
Cancer Prevention Programs	Prioritized actions to prevent cancer and other chronic diseases: o Over seven million pounds of healthy food have been distributed to families through pantries, mobile food fairs, and school-based programming o 73,000 + students have participated in school-based health and physical activity programs o 10,000 + students and staff have access to tobacco prevention resources through high school and colleges campuses o 24 vaccination clinics have been hosted to provide all recommended free shots to adolescents, including the HPV vaccine o 130,000 + residents have received direct support through Be Well Communities initiatives to date through participation in programs (e.g., after-school soccer, nutrition education classes, HPV vaccination mobile clinic visits, sun safety education) or access to infrastructure improvements (e.g., walking trails, sunshades, crosswalks)

### Community assessment phase and site selection: Acres Homes

In 2020, to further test and refine the effectiveness of our model, a rigorous community assessment was completed of communities in the Greater Houston Area, to prioritize potential implementation sites. The community assessment included understanding both community need and capacity. Community need included understanding demographic characteristics, health behaviors, and related outcomes, (e.g., rates of obesity, tobacco use, screening) as well as social, economic, and physical factors (e.g., median income, access to public transportation, available green space). Community capacity included understanding community assets, existing resources, ongoing initiatives, active coalitions, and key community partner organizations.

The assessment was also used to identify neighborhoods that refer in high numbers to Harris Health’s Lyndon B. Johnson (LBJ) Hospital, the safety net hospital where the MD Anderson Oncology Program provides cancer care for low-income and medically underserved residents. Selecting a site that is already directly connected to MD Anderson clinical services ensured that research, evidence-based community actions, and the delivery of clinical cancer care could more easily be integrated to overcome key challenges facing one of our local medically underserved communities. As a socio-culturally diverse community with both significant need and strong community resources, Acres Homes was selected as the next community to partner with to further test and refine the Be Well Communities model.

Acres Homes is a City of Houston-designated Super Neighborhood in northwest Houston with more than 57,000 residents [12]. This historic neighborhood was established during World War I and was once considered to be the American South’s largest unincorporated African-American community [13]. While the changing demographics show increasing numbers of residents of Hispanic descent (43% of the population), Acres Homes still experiences effects of a legacy of Blacks experiencing poverty. Table 2 includes further demographics for Acres Homes, including its status as a medically underserved area with relatively high rates of unhealthy behaviors, as compared to Healthy People 2030 goals. In Table 2, items that do not meet or exceed the Healthy People 2030 goals are indicated by bolded text. While the incidence of cancer is not higher in Acres Homes at this time, some specific modifiable risk factors for cancer are higher as compared to Harris County, the state of Texas and Healthy People 2030 goals. Importantly, cancer screening rates are on par with Harris County. However, rates of obesity and tobacco use are much higher in Acres Homes as compared to the county, state and the Healthy People 2030 goals. With that in mind, this cancer prevention initiative focused first on risk factors for obesity by attempting to meet or exceed the goals of Healthy People 2030. Specifically, 45% of residents are dealing with obesity, 18% are diabetic, and 36% self-described as sedentary outside of work [14]. An additional, ongoing challenge is that more than half of the population of Acres Homes resides in census tracts that are considered areas of persistent poverty [15]. Almost 40% of residents live under the federal poverty line, and 37% of adults are uninsured.

**Table 2:** Acres Homes demographics and key indicators related to cancer risk reduction compared with Healthy People 2030 goals. Items that do not meet or exceed Healthy People 2030 goals are targeted for improvement, as indicated by bolded text below

Total population (57,947)^a^	%				
Race					
White	14.1				
Black	44.9				
Hispanic	41.0				
Socioeconomic Indicator (age range)^b^	Acres Homes (%)				
Without Health Insurance (18 + years)	35.7				
Living below poverty level (all ages)	23.6				
Median household income	$41,358				
Health Indicator (age range)	Acres Homes (%)^b^	Harris County (%)^b^	Texas (%)^c^	U.S. (%)^c^	Healthy People 2030 Goal (%)^d^
Mammography use among women (50–74 years)	78.2	72.4	74.9	78.3	77.1
Papanicolaou smear use among women (21–65 years)	**81.2**	81.8	77.1	80.1	84.3
Up-to-date on colorectal cancer screening among adults (50–75 years)	**51.4**	55.4	59.8	69.9	74.4
Obesity among adults (18 + years)	**45.1**	33.0	34.8	30.9	36.0
No leisure-time physical activity among adults (18 + years)	**36.5**	28.0	25.6	23.8	21.2
Diabetes among adults (18 + years)	**17.6**	13.7	12.6	11.0	NA
Current smoking among adults (18 + years)	**21.2**	15.8	14.4	16.1	6.1
Cancer among adults (18 + years) *excluding skin cancer*	4.6	4.8	6.0	7.5	NA

^a^US Census Bureau: American Community Survey, 2014–2018

^B^Houston State of Health Survey, 2019

^c^Centers for Disease Control and Prevention, Behavior Risk Factor Surveillance System, 2018

^d^U.S. Department of Health and Human Services Healthy People 2030

NA – not available

Items that do not meet or exceed the Healthy People 2030 goals are indicated by bolded text.

While Acres Homes is a high-need community, it is also rich in assets, including actively engaged community members, organizations, and small businesses. In 2017, Acres Homes was selected as a part of the Mayor of Houston’s Complete Communities initiative [13]. Communities were selected by the city of Houston based on being under-resourced, having diverse demographic and physical characteristics, and maintaining a consistent level of civic engagement.

For each community selected, city of Houston leaders worked with residents and community organizations to develop an action plan focusing on a vision and specific goals to be carried out in a variety of areas, including health. For Acres Homes, the community prioritized four areas related to health: (1) increase access to fresh and healthy food, (2) nurture healthy and active living, (3) improve parks, trails, and community centers, and (4) improve well-being by expanding healthcare services and programs across the neighborhood. Through interviews and discussions with the Steering Committee formed during the community assessment phase, it was clear there was a strong desire to move forward with the health components of this plan, particularly as there had been little action since its creation in 2018. Specifically, there was an interest in doing work related to healthy eating and active living as the initial priority.

### Planning phase: Be Well™ Acres Homes

The planning phase of the project meant moving from the Community Assessment phase of the Be Well Communities model (Fig. 1) to launching Be Well™ Acres Homes and creating a new, health-focused Community Action Plan with organizations funded and activated to carry out EBIs. This process was launched in 2019. The following aims guided the planning phase:

- Aim 1: Convene community stakeholders (i.e. stakeholders as defined through an inclusive lens and leveled power structure lens as everyone with equal voice and agency in community decisions and community governance for health, including by not limited to residents, schools, nonprofit organizations, faith-based institutions, public and private entities, etc.) to share results of the Acres Homes Community Assessment, develop a common agenda, and facilitate strong community partnerships.
- Aim 2: Provide support and capacity building to community-based organizations in Acres Homes.
- Aim 3: Develop a Community Action Plan with the Be Well Acres Homes Steering Committee.

These aims were executed through convening monthly Be Well Acres Homes Steering Committee meetings, hosting trainings, and facilitating discussions with key stakeholders. Critical to the success of Be Well Acres Homes is fostering multi-sector collaboration through the steering committee and other meetings. See Table 3 for a summary of related meetings and topics covered.

**Table 3:** Be Well Acres Homes Meeting Timeline

Month	Topics	Attendees
September 2020	• Introduced the project and the Be Well Communities model including topics such as the collaborative approach, shared goals, target areas for interventions, utilization of evidence-based interventions, and focus on sustainability • Reviewed Steering Committee structure • Fostered trust and dialogue among all participants	32
October 2020	• Reviewed Community Assessment findings and validated results with attendees • Heard from a long-time resident about the history and opportunities for Acres Homes	45
November 2020	• Hosted presentations on current community programming related to healthy eating, healthcare open enrollment and COVID-19 testing and information	46
December 2020	• Hosted presentations on current community programming related to active living, gardening & community resources • Reviewed the Be Well Communities process for becoming a funded collaborating organization	53
January 2021	• Reviewed evidence-based interventions for implementation including the specific options available for consideration and prioritization • Hosted presentations on current community programming related to health outdoor initiatives and community health • Hosted collaborating organization training on selecting evidence-based interventions, developing objectives and evaluation measures, program planning, and timeline development	52
February 2021	• Hosted presentations on current community programming related to Safe Routes to School & community resources	63
March 2021	• Hosted presentations on current community programming related safety and physical activity	57
April 2021	• Reviewed Be Well Acres Homes proposals • Voted on proposals and Community Action Plan overall	69
May 2021	• Provided recap as well as additional information on Be Well Acres Homes proposals	56
June 2021	• Finalized Community Action Plan, based on voting and feedback • Reviewed Stakeholder Survey Pre-assessment results • Hosted presentation on current community programming related to gardening and public transportation • Sent out follow-up Stakeholder Survey in follow-up to meeting	57
July 2021	• Confirmed final Community Action Plan • Hosted community presentations and planned for the following year	50

*All meetings were hosted over the Zoom meeting platform due to the on-going COVID-19 pandemic*

## Methods

### Statistical methods

#### Aim1: convening & creating partnerships

The primary measure for partnership engagement over time among Steering Committee members was Stakeholder Surveys that were conducted at 2 time points (at the first meeting and 9 months later). The surveys provided data on four key indicators assessed for this phase of the project: (1) development of new community partnerships, (2) efficiency resulting from partnerships, (3) goal development and attainment, and (4) confidence that the Community Action Plan is representative of community priorities and needs. Surveys were administered by RTI International using an online platform in September 2020 to gather baseline data and again in June 2021 to assess progress after nearly a year. Participants rated most questions on a scale of 1 to 5. Additionally, they entered a number for the average number of partners they work with in Acres Homes. Subsequent stakeholder survey administrations are planned to occur on an annual basis to continue to assess the coalition function.

#### Aim 2: providing capacity building

The long-term sustainability of community-based interventions is supported by increasing capacity of community organizations to shape outcomes which requires, in turn, that the community organizations have the resources and tools necessary to implement and sustain EBIs. During the planning phase, the primary goal was to introduce the Steering Committee to EBIs that align with their organization’s mission and share a process for the collection and measurement of related metrics. Organizations interested in co-implementing EBIs with the community and becoming a funded collaborating organization were required to attend a two-hour training and submit a proposal for review. The training covered administrative items such as collaborating organization expectations and requirements as well as an overview of the proposal packet components and criteria for review. Other topics covered included introduction and selection of EBIs, implementation of effective programs, and program evaluation. Participants were invited to connect with each other to align proposal ideas and collaborate. Facilitated conversations were also hosted with organizations interested in similar EBI and topic areas, to coordinate potential proposals (or stimulate collaboration). In addition to the training, capacity building in the planning phase also includes activities such as identifying guest speakers to attend working groups to provide expertise on a topic of interest, conducting site visits to assist with program planning, and hosting facilitated group discussions with organizations interested in similar topics to foster collaboration and alignment.

Data related to the provision of capacity-building was tracked by assessing the number of organizations supported and total hours spent by the Be Well Communities team. Additionally, the Stakeholder Survey offered context to understand the role of assistance from the Be Well Communities team.

Of note, in the inaugural communities, organizations were directed to use robust tools such as the Community Guide [4] and Robert Wood Johnson Foundation’s (RWJF) What Works for Health [5] to prioritize and propose EBIs. Several organizations, especially those who do not focus primarily on health, requested more streamlined materials focused on cancer prevention that included summaries of the EBIs as well as metrics of success. As a part of this project we created and used a new resource to serve as a primer to support organizations in selecting EBIs that can be implemented with fidelity yet also tailored to the community [16].

#### Aim 3: developing the community action plan

The Community Action Plan is the foundation for community implementation and includes all information related to the EBIs to be implemented collaboratively. The Be Well Communities team led the Steering Committee through a series of meetings to develop the Community Action Plan in response to the desired focus on healthy eating and active living (Table 3).

Steering Committee organizations had the opportunity to submit a proposal to become a collaborating organization that would be funded to implement and sustain one or more EBIs to advance the community’s health. Proposals were competitively vetted by faculty and staff at MD Anderson to assess effectiveness and fiscal management as well as by RTI International to assess the alignment of the evaluation plan for each project with the overall evaluation plan for Be Well Communities. The Steering Committee and a panel of residents concurrently reviewed proposals, excluding their own submissions, to assess alignment with community priorities and needs as well as to ensure proposals represented new or enhanced programmatic activities. Additional information about each of these groups has been described previously [9]. Across all levels of review, proposals are assessed to determine if:

1. The defined project:
2. Aligns to the priorities and needs of the community and includes a plan to connect activities directly to community residents
3. Is an evidence-based intervention that will be implemented with fidelity but has been appropriately adapted for the community
4. Has SMART objectives and an evaluation plan with appropriate monitoring and evaluation metrics and associated data collection processes
5. Is not duplicative of work already underway in the community
6. Includes a clear problem statement and solution with a clear pathway to sustainability
7. The lead organization
8. Has the experience, capacity, resources, and buy-in from appropriate community leaders and other stakeholders to succeed and mitigate risks
9. Has the ability to measure impact
10. Can sustain this work after the initial funding ends

This project was approved by MD Anderson’s Quality Improvement Assessment Board. Informed consent was obtained when appropriate. The survey results were aggregated to maintain anonymity of respondents, and informed consent was obtained from participants.

## Results

The planning phase, focused on creating plans and partnerships, carried out from February 2019 through August 2021, successfully achieved all outcomes associated with each aim as described below.

### Aim 1: convening & creating partnerships

Across 15 Steering Committee meetings, convening an average of 53 attendees from more than 30 organizations, stakeholders reported progress across key metrics assessed over an initial 10-month interval (see Table 4).

**Table 4:** Results from the Stakeholder Survey at two time points in the initial planning process–September 2020 and June 2021

	Pre-planning Assessment (Sept 2020)		Year 1 Post-planning Assessment (June 2021)	
	N	Percentage	N	Percentage
1: Development of new partnerships in the community				
Average number of partners to carry out health-related programs and activities in Acres Homes	8	Mean 4.4 (Standard Deviation 3.1)	21	Mean 5.7 (Standard Deviation 3.4)
“We have developed new partnerships in the community through our participation in the Be Well Acres Homes Steering Committee.” (Rated from 1 = strongly disagree to 5 = strongly agree)			33/41	80.5% (agree and strongly agree)
2: Efficiency because of partnerships with other community organizations				
“My organization’s programs and activities will be delivered more efficiently and effectively because of partnerships with other community organizations in Acres Homes.” (Rated from 1 = strongly disagree to 5 = strongly agree)^**b**^	13/15	86.7% (agree and strongly agree)	33/41	80.5% (agree and strongly agree)
“We have connected with individuals who have positively impacted (or will positively impact) my organization’s work through our participation in the Be Well Acres Homes.” (Rated from 1 = strongly disagree to 5 = strongly agree)			35/41	85.4% (agree and strongly agree)
3: Initiative clear goals and goal achievement				
“Be Well Acres Homes has a clear written description of its goals.” (Rated from 1 = Does not describe this initiative at all, 2 = Describes this initiative a little, 3 = Somewhat describes this initiative, 4 = Mostly describes this initiative, 5 = Fully describes this initiative)			40/40	100% (mostly to fully describes)
“Overall, how confident are you that Be Well Acres Homes will achieve its goals?” (Rated from 1 = not at all confident to 5 = completely confident)	22/30	73.4% (very and completely confident)	32/41	78% (very and completely confident)
4: Confidence in the Community Action Plan’s development				
“The Community Action Plan for Be Well Acres Homes reflects the priorities of the community.” (Rated from 1 = strongly disagree to 5 = strongly agree)			38/41	93% (agree and strongly agree)
“The program activities described in the Community Action Plan will reach the residents most in need.” (Rated from 1 = strongly disagree to 5 = strongly agree)			38/41	93% (agree and strongly agree)

^a^ Gray boxes indicate a question was not asked in the pre-assessment survey

^b^ The wording of these questions varied slightly between the pre-assessment and Year 1 surveys. The question wording in the table comes from the Year 1 survey. The pre-assessment survey question was worded as (differences are in bold): “My organization’s programs and activities are delivered more efficiently because of partnerships with other community organizations in Acres Homes.”

The survey to measure collective impact and partnership engagement in September 2020 included 30 Steering Committee members. At follow-up in June 2021, some additional items about progress to that point were added, and 41 Steering Committee members participated. Participants in the two surveys included some but not all the same people at each time point, representing the same organizations as well as new organizations that joined over time.

Stakeholders reported that they are developing new partners in the community (80.5%), working together more effectively (80.5%), and connecting with others in ways that will lead to a positive impact as a result of this initiative (85.4%). Additionally, a one-tailed paired-sample t-test was performed to compare the new number of partners in June 2021 against the baseline assessed in September 2020. The mean value of organization partners to carry out health-related programs (M = 5.7, SD = 3.4) was significantly higher than the baseline mean of 4.4 [t(20) = 1.752, *p* = 0.047]. The data show a noteworthy growth in collaborative work, as also demonstrated by the other data points from June 2021 and the qualitative comments noted below (see Table 4).

Further, stakeholders reported appreciating that the initiative shows clear goals (100%) and expressed increased confidence that goals will be achieved. A one-tailed paired-sample t-test was performed to compare participant perceptions of overall confidence that Be Well Acres Homes will achieve its goals. The mean value from the 1–5 response index in June (M = 4, SD = 0.7) was significantly higher than in the original data collection 10 months earlier [t(40) = 1.829, *p* = 0.037], showing belief that the effort will be successful.

Qualitatively, stakeholders responded in a similar manner, as one respondent noted, Be Well Acres Homes is “one of the most effective collaboratives I have ever engaged with. Extremely well led, great and thorough communication. Goals are clear and emphasized repeatedly.”

Perhaps most importantly, in June 2021, after 10 months of collaboration, stakeholders felt confident that the Community Action Plan reflects the priorities of the community (93%) and will reach the residents most in need (93%).

On one measure, there was a decrease in stakeholders’ responses that programs will be delivered more efficiently and effectively because of partnerships with other community organizations. However, this item was still rated quite highly with more than 80% agreeing or strongly agreeing that programs would be delivered more effectively and efficiently because of this coalition. While it is an area to continue monitoring to ensure it is not an unfavorable trend, it is possible this result is affected by going from fewer respondents (15) to a much larger number (41) and also because of the question being presented in a double-barreled manner, inquiring both about effectiveness and efficiency, since those goals can be at odds in coalitions. Future versions of the survey will be edited to separate these items to better understand collaborators’ perspectives.

### Aim 2: providing capacity building

Throughout the project period, the Be Well Communities team provided more than 400 hours of capacity building support to 30 community-based organizations which included leading a training session of effective implementation and evaluation of EBIs, Steering Committee meetings and working groups which covered topics of specific interest to the organizations, and individual and group meetings with organizations to discuss tailoring and sustaining specific EBIs to be carried out by each organization. For those organizations that participated in all activities hosted by the Be Well Communities team, 91% ranked assistance from MD Anderson as a top factor in developing a project plan to be implemented as a part of the Community Action Plan. Additionally, 50 organizations that participated across all implementation sites for Be Well Communities received the primer developed for implementing EBIs in cancer prevention.

### Aim 3: development of the community action plan

The Be Well Acres Homes Community Action Plan was developed with and for the community; 18 organizations, including neighborhood schools, community clinics, city parks and recreation department, and food pantries, are implementing 15 EBIs focused on active living and healthy eating (see Table 5). These projects are guided by the Steering Committee to ensure resident feedback and long-term sustainability are built into all aspects of the work. As a place-based initiative, all residents of Acres Homes can participate in Be Well Acres Homes active living and healthy eating interventions and investments. The Community Action Plan builds on the roadmap developed through the City of Houston Mayor’s Office Complete Communities [13]; and addresses the four prioritized health related areas requested by the community.

**Table 5:** Be Well Acres Homes Community Action Plan’s Evidence-based Interventions

Evidence-based intervention	Anticipated long-term health outcomes (5–10 years)	Acres Homes lead implementer(s)
Active living		
Community fitness programs ^a^	Increased physical activity Improved physical fitness Improved health outcomes	HHD, HPARD
Community-Based Social Support for Physical Activity ^a,b^	Increased physical activity Improved physical fitness Improved health outcomes	HPARD, HPB, HHD
Exercise Prescriptions ^a^	Increased physical activity Improved physical fitness Increased mobility	HHS
Individually-Adapted Physical Activity Programs ^a^	Increased physical activity Improved physical fitness	HHS
Physically Active Classrooms ^a,b^	Increased physical activity	Aldine ISD
Places for Physical Activity ^a,b^	Increased physical activity Improved physical fitness	Aldine ISD, HPARD, HHD, HHS
Safe Routes to School ^a,b,c^	Increased physical activity Improved health outcomes	HCPH with support from COH
School-based Physical Education Enhancements ^a,b,c,^	Increased physical activity Improved physical fitness	Aldine ISD
Healthy eating		
Fruit & Vegetable Incentive Programs ^a^	Increased access to healthy food Increased healthy food purchases Increased fruit and vegetable consumption	BB
School Fruit and Vegetable Gardens ^a,b^	Increased willingness to try fruits and vegetables Increased fruit and vegetable consumption	Multiple
Multi-Component and combined efforts		
Community Health Workers ^b^	Increased patient knowledge Increased access to care Increased healthy behaviors Increased preventive care Increased screening for breast, cervical, colorectal cancers	HHS
Multi-Component Obesity Prevention Interventions ^a^	Increased physical activity Improved weight status	All
Multi-Component School-Based Obesity Prevention Interventions ^a,b^	Increased physical activity Improved weight status Improved dietary habits	Multiple
Nutrition & Physical Activity Interventions in Preschool & Child Care ^a,b,c^	Improved nutrition Increased physical activity Improved weight status	HCPH
Screen Time Interventions for Children ^a,b,^	Reduced sedentary screen time Increased physical activity Improved dietary habits Improved weight status	Aldine ISD

^a^ Robert Wood Johnson Foundation. (2019). What Works for Health Guide. Retrieved 19 July 2021

^b^ Community Preventive Services Task Force. The Guide to Community Preventive Services (The Community Guide). US Department of Health and Human Services. Accessed 19 July 2021

^c^ Centers for Disease Control and Prevention. High Impact in 5-Years. (2022). Centers for Disease Control and Prevention Office of Policy Analytics and Population Health. Accessed 15 November 2022

Beauty’s Community Garden (BCG); Brighter Bites (BB); City of Houston (COH); Harris County Public Health (HCPH); Harris Health System (HHS); Houston Health Department (HHD); Houston Parks and Recreation Department (HPARD); Houston Parks Board (HPB); Prairie View A&M University (PVAMU)

Initial activities included:

- expanding exercise and healthy eating programs for all ages
- building community and school-based gardens
- increasing access to fresh, healthy food
- improving parks, trails, the community center, and places for physical activity.
- expanding access to health care through a community health worker

The evaluation used a mixed methods approach, including qualitative data to understand the “why” and “how” that is reported in the quantitative data. Evaluation of the EBIs is based on the RE-AIM planning and evaluation framework, as it is a well-established framework for planning and systematically evaluating community-based programs [17, 18]. More than 400 metrics are being reported quarterly and synthesized annually in accordance with the evaluation plan to determine the success of implementation and process measures that will indicate trends toward achievement of the anticipated long-term health outcomes (5–10 years) concordant with each EBI (outlined in Table 5). Outcomes related to these EBIs such as increased physical activity and fruit and vegetable consumption are associated with decreasing the risk factors linked to obesity.

## Discussion

Existing research makes clear that correcting social, economic, and health inequities has the most significant impact in improving cancer outcomes [20], a theme supported as long ago as the late 1800s with research noting that social factors, not genetics, drive many racial disparities [19, 21]. Disparities in cancer outcomes are often reduced after controlling for racial differences in socioeconomic status [20], as a range of studies show that if individuals have equal access to quality care, health outcomes can be improved among different racial groups [22–24]. While Acres Homes is unique, the story of the community is not in that it mirrors many communities of color across the city of Houston and the country. Beyond worse health outcomes and community disinvestment, erosion of trust in institutions meant to address community needs is pervasive. These factors emphasize the substantial barriers that persist in accessing healthy food, active recreation, and health attainment, all acutely relevant here because access to and the consumption of healthy food are inextricably linked to obesity which is associated with an increased risk for thirteen cancer types [1]. To reduce barriers to resources and thus cancer risks, it is critical to empower multi-sectoral community-based organizations—such as schools, community colleges, non-profit organizations, workplaces, government agencies, and policymakers with proven, long-term strategies (i.e., EBIs), especially in communities with limited resources. A collaborative approach driven by the wants and needs of the community facilitates an effective infrastructure that promises to improve cancer-related outcomes in the community over the long term.

By focusing on the needs of the community to engage residents and long-time community advocates on the Steering Committee guiding this work, the Community Action Plan both reflects the priorities of the community and is well-positioned to reach the residents most in need while serving as a model for how other communities can co-create progress. While this manuscript reports on the early phase of implementation, the key components for effective community-based approaches are being put into place:

- (1) Making health equity a shared vision and value

The Steering Committee is working towards a shared vision through well-defined goals. By working together, the Steering Committee is well positioned to collectively achieve these goals.

- (2) Fostering multi-sector collaboration

The important first steps of collaboration necessary for a long-term collaborative future are taking shape. Community organizations and residents are developing new partners and connecting with others in ways that will lead to a positive impact as a result of this approach.

- (3) Increasing community capacity to shape health outcomes

Community organizations that will be in Acres Homes for the long term were supported to develop strategic plans to carry out specific EBIs in the Community Action Plan that can improve health. The Community Action Plan addresses historical inequities in the community by building food access though community and school gardens; increasing safe spaces for active transportation and recreation; and countering barriers associated with helpful resources that can reduce cancer risk factors (e.g., providing a community health worker, offering programming onsite at the health clinic, engaging in the locations where people are active such as schools and churches). Over the next several years, the Be Well Communities team will continue to work with organizations throughout the implementation phase increasing the capacity of the community to shape health outcomes and to ensure the long-term sustainability of this work.

## Conclusions

Enthusiasm is building in Acres Homes, and MD Anderson is privileged to guide this initiative in partnership with the community now and in the years to come, supported by both the CCSG COE mandate and the opportunity that the P30 administrative supplement afforded for this project. Through its Cancer Prevention & Control Platform and the Be Well Communities model, MD Anderson’s novel and sustained commitment to strengthening the systems that underlie the achievement of good health provided an opportunity to enhance an effective model for community impact in a manner that works toward justice through a focus on addressing health inequities in one of Houston’s most historic yet disinvested communities.

## Data Availability

The data underlying this article will be shared on reasonable request to the corresponding author.
